# Assessment of the ABC_2_-SPH risk score to predict invasive mechanical ventilation in COVID-19 patients and comparison to other scores

**DOI:** 10.3389/fmed.2023.1259055

**Published:** 2023-11-16

**Authors:** Christiane Corrêa Rodrigues Cimini, Polianna Delfino-Pereira, Magda Carvalho Pires, Lucas Emanuel Ferreira Ramos, Angélica Gomides dos Reis Gomes, Alzira de Oliveira Jorge, Ariovaldo Leal Fagundes, Bárbara Machado Garcia, Bruno Porto Pessoa, Cíntia Alcantara de Carvalho, Daniela Ponce, Danyelle Romana Alves Rios, Fernando Anschau, Flavia Maria Borges Vigil, Frederico Bartolazzi, Genna Maira Santos Grizende, Giovanna Grunewald Vietta, Giulia Maria dos Santos Goedert, Guilherme Fagundes Nascimento, Heloisa Reniers Vianna, Isabela Muzzi Vasconcelos, Joice Coutinho de Alvarenga, José Miguel Chatkin, Juliana Machado Rugolo, Karen Brasil Ruschel, Liege Barella Zandoná, Luanna Silva Monteiro Menezes, Luís César de Castro, Maíra Dias Souza, Marcelo Carneiro, Maria Aparecida Camargos Bicalho, Maria Izabel Alcântara Cunha, Manuela Furtado Sacioto, Neimy Ramos de Oliveira, Pedro Guido Soares Andrade, Raquel Lutkmeier, Rochele Mosmann Menezes, Antonio Luiz Pinho Ribeiro, Milena Soriano Marcolino

**Affiliations:** ^1^Hospital Santa Rosália, Teófilo Otoni, Minas Gerais, Brazil; ^2^Mucuri's Medical School and Telehealth Center, Universidade Federal dos Vales do Jequitinhonha e Mucuri (UFVJM), Teófilo Otoni, Minas Gerais, Brazil; ^3^Universidade Federal de Minas Gerais and Institute for Health and Technology Assessment (IATS), Porto Alegre, Rio Grande do Sul, Brazil; ^4^Department of Statistics, Universidade Federal de Minas Gerais, Belo Horizonte, Minas Gerais, Brazil; ^5^Hospitais da Rede Mater Dei, Belo Horizonte, Minas Gerais, Brazil; ^6^Hospital Risoleta Tolentino Neves, Belo Horizonte, Minas Gerais, Brazil; ^7^Hospital Universitário de Santa Maria, Santa Maria, Rio Grande do Sul, Brazil; ^8^Faculdade de Ciências Médicas de Minas Gerais, Belo Horizonte, Minas Gerais, Brazil; ^9^Hospital Júlia Kubitschek, Belo Horizonte, Minas Gerais, Brazil; ^10^Hospital João XXIII, Belo Horizonte, Minas Gerais, Brazil; ^11^Hospital das Clínicas da Faculdade de Medicina de Botucatu, Av. Prof. Mário Rubens Guimarães Montenegro, UNESP, Botucatu, São Paulo, Brazil; ^12^Universidade Federal de São João del-Rei, Divinópolis, Minas Gerais, Brazil; ^13^Hospital Nossa Senhora da Conceição and Hospital Cristo Redentor, Porto Alegre, Rio Grande do Sul, Brazil; ^14^Hospital Metropolitano Dr. Célio de Castro, Belo Horizonte, Minas Gerais, Brazil; ^15^Hospital Santo Antônio, Curvelo, Minas Gerais, Brazil; ^16^Hospital Santa Casa de Misericórdia de Belo Horizonte, Belo Horizonte, Minas Gerais, Brazil; ^17^Hospital SOS Cárdio, Florianópolis, Santa Catarina, Brazil; ^18^Universidade Federal de Santa Maria, Santa Maria, Rio Grande do Sul, Brazil; ^19^Hospital Unimed BH, Belo Horizonte, Minas Gerais, Brazil; ^20^Hospital Universitário Ciências Médicas, Belo Horizonte, Minas Gerais, Brazil; ^21^Department of Internal Medicine, Medical School, Universidade Federal de Minas Gerais, Belo Horizonte, Minas Gerais, Brazil; ^22^Telehealth Center, University Hospital, Universidade Federal de Minas Gerais, Belo Horizonte, Minas Gerais, Brazil; ^23^Hospital João XXIII, Belo Horizonte, Minas Gerais, Brazil; ^24^Hospital São Lucas PUCRS, Porto Alegre, Rio Grande do Sul, Brazil; ^25^Pontifica Universidade Católica do Rio Grande do Sul, Porto Alegre, Rio Grande do Sul, Brazil; ^26^Institute for Health Technology Assessment (IATS/CNPq), Porto Alegre, Rio Grande do Sul, Brazil; ^27^Hospital Mãe de Deus, Porto Alegre, Rio Grande do Sul, Brazil; ^28^Hospital Universitário de Canoas, Canoas, Rio Grande do Sul, Brazil; ^29^Hospital Bruno Born, Lajeado, Rio Grande do Sul, Brazil; ^30^Hospital Metropolitano Odilon Behrens, Belo Horizonte, Minas Gerais, Brazil; ^31^Hospital Santa Cruz, Santa Cruz do Sul, Rio Grande do Sul, Brazil; ^32^Fundação Hospitalar do Estado de Minas Gerais (FHEMIG), Cidade Administrativa de Minas Gerais, Belo Horizonte, Minas Gerais, Brazil; ^33^Centro Universitário de Belo Horizonte (UNIBH), Belo Horizonte, Minas Gerais, Brazil; ^34^Hospital Eduardo de Menezes, Belo Horizonte, Minas Gerais, Brazil; ^35^Telehealth Center, University Hospital, Universidade Federal de Minas Gerais, Belo Horizonte, Minas Gerais, Brazil; ^36^Cardiology Service, University Hospital, Universidade Federal de Minas Gerais, Belo Horizonte, Minas Gerais, Brazil; ^37^Department of Internal Medicine, Medical School and University Hospital, Universidade Federal de Minas Gerais, Belo Horizonte, Minas Gerais, Brazil; ^38^Institute for Health Technology Assessment (IATS), Porto Alegre, Rio Grande do Sul, Brazil

**Keywords:** COVID-19, intensive care unit, prognosis, invasive mechanical ventilation, risk assessment

## Abstract

**Background:**

Predicting the need for invasive mechanical ventilation (IMV) is important for the allocation of human and technological resources, improvement of surveillance, and use of effective therapeutic measures. This study aimed (i) to assess whether the ABC_2_-SPH score is able to predict the receipt of IMV in COVID-19 patients; (ii) to compare its performance with other existing scores; (iii) to perform score recalibration, and to assess whether recalibration improved prediction.

**Methods:**

Retrospective observational cohort, which included adult laboratory-confirmed COVID-19 patients admitted in 32 hospitals, from 14 Brazilian cities. This study was conducted in two stages: (i) for the assessment of the ABC_2_-SPH score and comparison with other available scores, patients hospitalized from July 31, 2020, to March 31, 2022, were included; (ii) for ABC_2_-SPH score recalibration and also comparison with other existing scores, patients admitted from January 1, 2021, to March 31, 2022, were enrolled. For both steps, the area under the receiving operator characteristic score (AUROC) was calculated for all scores, while a calibration plot was assessed only for the ABC_2_-SPH score. Comparisons between ABC_2_-SPH and the other scores followed the Delong Test recommendations. Logistic recalibration methods were used to improve results and adapt to the studied sample.

**Results:**

Overall, 9,350 patients were included in the study, the median age was 58.5 (IQR 47.0–69.0) years old, and 45.4% were women. Of those, 33.5% were admitted to the ICU, 25.2% received IMV, and 17.8% died. The ABC_2_-SPH score showed a significantly greater discriminatory capacity, than the CURB-65, STSS, and SUM scores, with potentialized results when we consider only patients younger than 80 years old (AUROC 0.714 [95% CI 0.698–0.731]). Thus, after the ABC_2_-SPH score recalibration, we observed improvements in calibration (slope = 1.135, intercept = 0.242) and overall performance (Brier score = 0.127).

**Conclusion:**

The ABC_2_-SPHr risk score demonstrated a good performance to predict the need for mechanical ventilation in COVID-19 hospitalized patients under 80 years of age.

## Highlights

- Among 9,3,150, 33.5% were admitted to the ICU, 25.2% received IMV, and 17.8% died.
- Patients who received IMV had higher median age and prevalence of hypertension and diabetes.
- ABC_2_-SPH score presented poor discrimination and calibration, with better discrimination among patients <80 years.
- In patients <80 years, the score had greater discrimination ability than CURB-65, SOFA, STSS and SUM scores.
- After the recalibration, ABC_2_-SPHr score obtained better calibration and overall performance.

## Background

Since its inception, the COVID-19 pandemic has triggered an unprecedented crisis in health systems worldwide, with increased demand for intensive care unit (ICU) beds and mechanical ventilation (1). Although studies highlight the substantial impact of vaccination on the trajectory of the pandemic, with up to 90% protection against COVID-19-associated invasive mechanical ventilation (IMV) and death among adults (2, 3). It is estimated that the mortality rate associated with IMV continues overcoming 30% (4). A recent systematic review and meta-analysis found a 43% (95% CI 0.29–0.58) pooled IMV mortality rate (1). Knowledge of COVID-19 intensive care unit (ICU) and associated IMV patient characteristics, and outcomes as well as analyzing their regional variability is critically important for patient management and allocation of resources (1). Therefore, it may be helpful to predict which patients are more likely to progress to IMV, to subsidize more assertive health decisions.

Although different prognostic scores have been proposed to predict IMV among COVID-19 patients, the majority of them present methodological limitations, restricting their clinical applicability (for more details, see Supplementary Table S1). Furthermore, most scores were developed in high-income countries, without external validation in low-and middle-income countries.

In this context, the ABC_2_-SPH risk score for predicting in-hospital mortality was rigorously developed and validated in Brazilian patients with high discrimination (5). This score is the only mortality risk score for COVID-19 tested and validated in the Brazilian population (5). It predicts in-hospital mortality in patients with COVID-19 using easily accessible variables on admission: **A**ge, **B**UN (blood urea nitrogen), **C**omorbidities, **C**-reactive protein, **S**pO_2_/FiO_2_ ratio, **P**latelet count, and **H**eart rate. The score ranges from 0 to 20, with the following risk groups: low (0–1), intermediate (2–4), high (5–8), and very high (≥9). It is freely available as an online risk calculator.^1^ It was developed in a cohort of 3,978 patients admitted to 36 hospitals in five Brazilian states. The validation was carried out on 1,054 patients admitted to the same institutions (temporal validation) and also in a cohort with 474 Spanish patients (external validation). It has shown good overall performance for temporal (AUROC = 0.859 [95% CI 0.833 to 0.885], Brier = 0.108 and calibration [slope = 1.138, intercept = 0.114, value of *p* = 0.184]) and external validation (AUROC = 0.894 [95% CI 0.870 to 0.919] Brier = 0.093) (5). However, evidence of its accuracy for IMV prediction is still lacking. Therefore, our aims were: (i) to assess whether the ABC_2_-SPH score is able to predict IMV in COVID-19 patients; (ii) to compare its performance with other existing scores; (iii) to perform score recalibration, and to assess whether recalibration improved prediction.

## Methods

### Study design

This study is a substudy of the retrospective multicenter cohort Brazilian COVID-19 Registry, conducted in 32 Brazilian hospitals, in 14 cities from five Brazilian states (Minas Gerais, Pernambuco, Rio Grande do Sul, Santa Catarina and São Paulo), described in detail elsewhere (6). The study was approved by the National Commission for Research Ethics (CAAE 30350820.5.1001.0008) and the individual informed consent was waived due to the pandemic circumstances and analysis of unidentified data.

### Study population

The cohort study included consecutive adult patients (≥18 years-old) with laboratory-confirmed COVID-19, according to World Health Organization guidance (7), admitted in one of the participating hospitals. For the assessment of the ABC_2_-SPH score and the comparison with other scores, patients admitted from July 31, 2020, to March 31, 2022, were included. For ABC_2_-SPH score recalibration and also comparison with other scores, patients admitted from January 1, 2021, to March 31, 2022, were enrolled. However, for recalibration, only patients younger than 80 years were included, since mortality is particularly high for mechanical ventilation at an older age. This supported recommendations for conservative treatment for elderly and/or frail patients (8–10).

**Figure 1:**
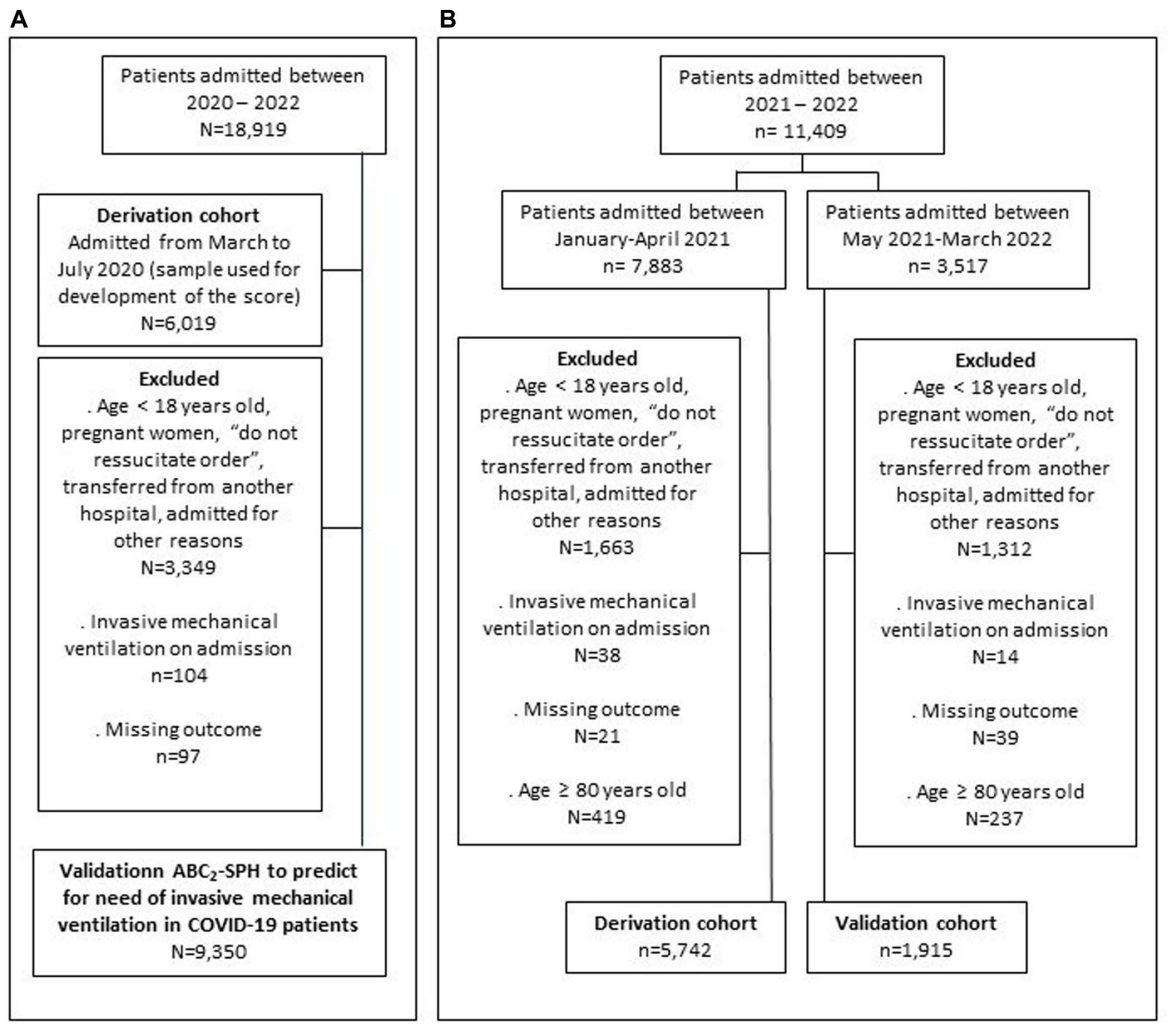
Flowchart of the study conducted in two stages: **(A)** The first stage, aimed to assess the ABC_2_-SPH risk score to predict invasive mechanical ventilation in COVID-19 patients and compare with other available scores; and **(B)** the second stage, aimed to perform ABC_2_-SPH score recalibration, as well as to compare with other scores.

Patients with at least one of the following conditions were excluded: (i) pregnant women; (ii) “do not resuscitate” order; (iii) patients who manifested COVID-19 while admitted for other conditions; (iv) those transferred to other hospitals who had no defined outcome (discharged or death); (v) patients who were already on IMV at hospital presentation; and (vi) exclusively for score recalibration, patients ≥80 years old (Figure 1).

### Data collection

Medical records were reviewed to collect data concerning the patients’ characteristics, including age, sex, pre-existing comorbid medical conditions and medications taken at home; COVID-19-associated symptoms at hospital presentation; clinical assessment upon hospital presentation; laboratory results; inpatient medication, treatment, and outcomes. The data collection instrument was designed with reference to COVID-19 guidelines from the World Health Organization and the Brazilian Ministry of Health, as previously described (6).

A detailed guidance manual for data collection was developed, containing the definitions used in the study (Supplementary material). It was provided to all participating centers, and online training was mandatory before local research personnel were allowed to start collecting study data.

Data was collected by trained researchers from the medical records, using Research Electronic Data Capture (REDCap®) (version 7.3.1) (11, 12), hosted at the Telehealth Center of the University Hospital, of the *Universidade Federal de Minas Gerais* (13). To ensure reliability and monitor data, a code was developed in the R software that periodically verified possible data entry errors. When detected, the analysts notified the participating center for correction.

### Outcomes

The primary outcome was IMV during hospitalization.

### Sample size

Model validation followed guidance from the Transparent Reporting of a Multivariable Prediction Model for Individual Prediction or Diagnosis (TRIPOD) checklist (14, 15) and the Prediction model Risk Of Bias Assessment Tool (PROBAST) (16). TRIPOD checklist ideally recommends at least 100 events (as deaths) and 100 non-events as samples for score validation. In the present analysis, the sample size was not calculated, since all patients eligible by the inclusion criteria were enrolled.

### Statistical analysis

Continuous variables were described by medians and interquartile ranges (IQR) and categorical variables were represented by absolute and relative frequencies. Data were analyzed with R software (version 4.0.2), using mice (function mice), pROC (functions roc, ci.auc, roc.test), glmnet (function cv.glmnet), tidyverse (dplyr functions), gtsummary (function tbl_summary) and ggplot2 packages. value of *p*s <0.05 were considered statistically significant.

The statistical analysis was divided into two stages: (i) evaluation of the ABC_2_-SPH risk score for predicting IMV in COVID-19 patients and comparison with other available scores; and (ii) recalibration of the ABC_2_-SPH score, as well as comparison with other scores.

### ABC_2_-SPH assessment and comparison with other risk scores

Discrimination of the ABC_2_-SPH score was compared to other existing scores, including CALL (17), COVID-IRS (18), CURB-65 (19), PREDI-CO (20), SOFA (21), STSS (22), SUM (23) and 4C Mortality Score (24). The scores were chosen based on the two conditions: (i) parameters available within the Registry’s database, and (ii) accessible methods for calculation.

The main characteristics of the scores are listed in Supplementary Table S1. The comparison of ABC_2_-SPH (5) with other scores (17–24) was performed using the number of complete cases for each score (non-imputed database) through a procedure for unpaired receiving operator characteristic (ROC) curves that is an extension of Delong et al. recommendations (25). This procedure was implemented in the pROC package, function “roc.test.” Due to the multiple comparisons, alpha was corrected using the Bonferroni method.

### Score recalibration

The score was recalibrated, in an attempt to improve the prediction risk of IMV among patients with COVID-19. The sample of patients included COVID-19 patients under 80 years of age, divided into derivation (from January 1 to April 30, 2021) and validation cohorts (from May 1, 2021, to March 31, 2022), resulting in approximately 75 and 25% of the sample, respectively. This division guarantees the minimum of 100 events in the validation cohort, as recommended by the TRIPOD checklist (14, 15).

The recalibration methods consisted of fitting a logistic regression model [for more details, see Steyerberg et al. (26)] in the derivation sample and the evaluation of the method was done in the validation sample.

### Missing data

To handle missing values, multiple imputation by chained equations (MICE) was used, considering missing at random assumption. The imputation technique included all variables with up to 30% missing values. The prediction of missing values was performed using all variables included in the analysis. Invasive mechanical ventilation was not imputed, and was not used as a predictor in the MICE model in the validation dataset. The predictive mean matching (PMM) method was used for continuous predictors and polytomous regression for categorical variables. Ten imputed datasets were obtained with 10 iterations, and their results were combined following Rubin’s rules (27).

### Performance measures

Model’s discrimination was assessed by the area under the ROC curve (AUROC), with 95% confidence interval (95% CI) calculated by bootstrap resampling, through 2,000 samples. A value of 0.5 indicates no predictive ability, 0.60 to 0.69 is considered poor, 0.70 to 0.89 good, and 0.90 to 1.0 excellent (28).

The accuracy of the predictive model was assessed using the Brier score, a measure that quantifies how close predictions are to the truth (29). The score ranges between 0 and 1, in which smaller values indicate superior model performance. Results were stratified by age groups (<60, 60–69, 70–79 and ≥ 80 years-old), sex and presence or absence of key comorbidities before recalibration, to assess score performance in different subgroups.

Calibration was assessed graphically by plotting the predicted IMV probabilities against the observed IMV, testing intercept equals zero and slope equals one, simultaneously.

## Results

### ABC_2_-SPH assessment and comparison with other risk scores

Overall, 9,350 patients were included in the study, the median age was 58.5 (IQR 47.0–69.0) years old, and 45.4% were women. Of those, 33.5% were admitted to the ICU, 25.2% received IMV, and 17.8% died. Patients who received IMV were older; had a higher frequency of hypertension, diabetes, obesity, chronic kidney disease, rheumatologic disease and previous transplant; a higher number of comorbidities; and a higher frequency of ICU, dialysis, thromboembolism and mortality, when compared to those who did not receive IMV (Table 1). They also had a higher frequency dyspnea, cough, fever, nausea, and arthralgia; clinical findings such as fever, tachycardia and arterial hypotension (Supplementary Table S4) and laboratory findings such as neutrophilia, lymphopenia, thrombocytopenia and increased lactate, D-dimer and C-reactive protein, when compared to those who did not receive IMV (Supplementary Tables S4, S5).

**Table 1:** Demographic data, clinical characteristics, and outcomes of a cohort of Brazilian patients admitted to hospital with COVID-19, from July 31, 2020, to March 31, 2022.

Characteristics	Overall *N* = 9,350^1^	Non-missing cases	IMV *N* = 2,361^1^	No IMV *N* = 6,989^1^	*p*-Value^2^
Age (years)	58.5 (47.0, 69.0)	9,350 (100%)	62.0 (51.0, 71.0)	57.0 (46.0, 68.0)	<0.001
Women	4,241 (45.4%)	9,350 (100%)	1,021 (43.2%)	3,220 (46.1%)	0.018
Comorbidities					
Hypertension	4,874 (52.1%)	9,350 (100%)	1,413 (59.8%)	3,461 (49.5%)	<0.001
Heart failure	373 (4.0%)	9,350 (100%)	119 (5.0%)	254 (3.6%)	0.003
Atrial fibrillation	374 (4.0%)	9,350 (100%)	118 (5.0%)	256 (3.7%)	0.005
COPD	388 (4.1%)	9,350 (100%)	107 (4.5%)	281 (4.0%)	0.309
Asthma	525 (5.6%)	9,350 (100%)	149 (6.3%)	376 (5.4%)	0.100
Diabetes mellitus	2,415 (25.8%)	9,350 (100%)	777 (32.9%)	1,638 (23.4%)	<0.001
Obesity	1,835 (19.6%)	9,350 (100%)	611 (25.9%)	1,224 (17.5%)	<0.001
Chronic kidney disease	331 (3.5%)	9,350 (100%)	119 (5.0%)	212 (3.0%)	<0.001
Cancer	235 (2.5%)	9,350 (100%)	64 (2.7%)	171 (2.4%)	0.527
Rheumatologic disease	178 (1.9%)	9,350 (100%)	64 (2.7%)	114 (1.6%)	0.001
Cirrhosis	24 (0.3%)	9,350 (100%)	8 (0.3%)	16 (0.2%)	0.498
Previous transplant	72 (0.8%)	9,350 (100%)	34 (1.4%)	38 (0.5%)	<0.001
HIV infection	68 (0.7%)	9,350 (100%)	17 (0.7%)	51 (0.7%)	>0.999
Comorbidities (total number)					
0	3,116 (33.3%)	9,350 (100%)	567 (24.0%)	2,549 (36.5%)	<0.001
1	2,942 (31.5%)	9,350 (100%)	750 (31.8%)	2,192 (31.4%)	
2	2,179 (23.3%)	9,350 (100%)	652 (27.6%)	1,527 (21.8%)	
3	905 (9.7%)	9,350 (100%)	323 (13.7%)	582 (8.3%)	
≥4	208 (2.2%)	9,350 (100%)	69 (3.0%)	139 (2.0%)	
Clinical outcomes					
ICU	3,124 (33.5%)	9,334 (100%)	2,261 (95.8%)	863 (12.4%)	<0.001
Dialysis	899 (9.6%)	9,344 (100%)	847 (36.0%)	52 (0.7%)	<0.001
Venous thromboembolism	462 (4.9%)	9,349 (100%)	200 (8.5%)	262 (3.7%)	<0.001
In-hospital mortality	1,665 (17.8%)	9,345 (100%)	1,509 (64.0%)	156 (2.2%)	<0.001

^1^Numbers are presented as *n* (%) or median (IQR). ^2^Statistical tests performed: chi-square test of independence; Wilcoxon rank-sum test; Fisher’s exact test. COPD: chronic obstructive pulmonary disease; HIV: human immunodeficiency virus; ICU: intensive care unit.

The AUROC for the ABC_2_-SPH 0.677 (0.661–0.694), and the Brier score 0.196. Subject-specific risks were calculated, and patients were classified according to ABC_2_-SPH risk groups (Table 2). Score’s performance was worse among older patients, especially the octogenarians, and patients with chronic pulmonary obstructive disease (Supplementary Table S1).

**Table 2:** Predicted and observed invasive mechanical ventilation (IMV) rates observed with ABC_2_-SPH score.

Risk groups	Score	Predicted IMV rate	Number of patients classified in each risk group	Number of IMV patients	Observed rate of IMV
Low	0–1	<6.0%	2,108 (22.5%)	183	8.7%
Intermediate	2–4	6.0–14.9%	3,489 (37.3%)	634	18.2%
High	5–8	15–49.9%	2,970 (31.8%)	1,115	37.5%
Very high	≥ 9	≥50%	783 (8.4%)	429	54.8%
Overall	-	-	9,350 (100%)	2,361	25.3%

**Figure 2:**
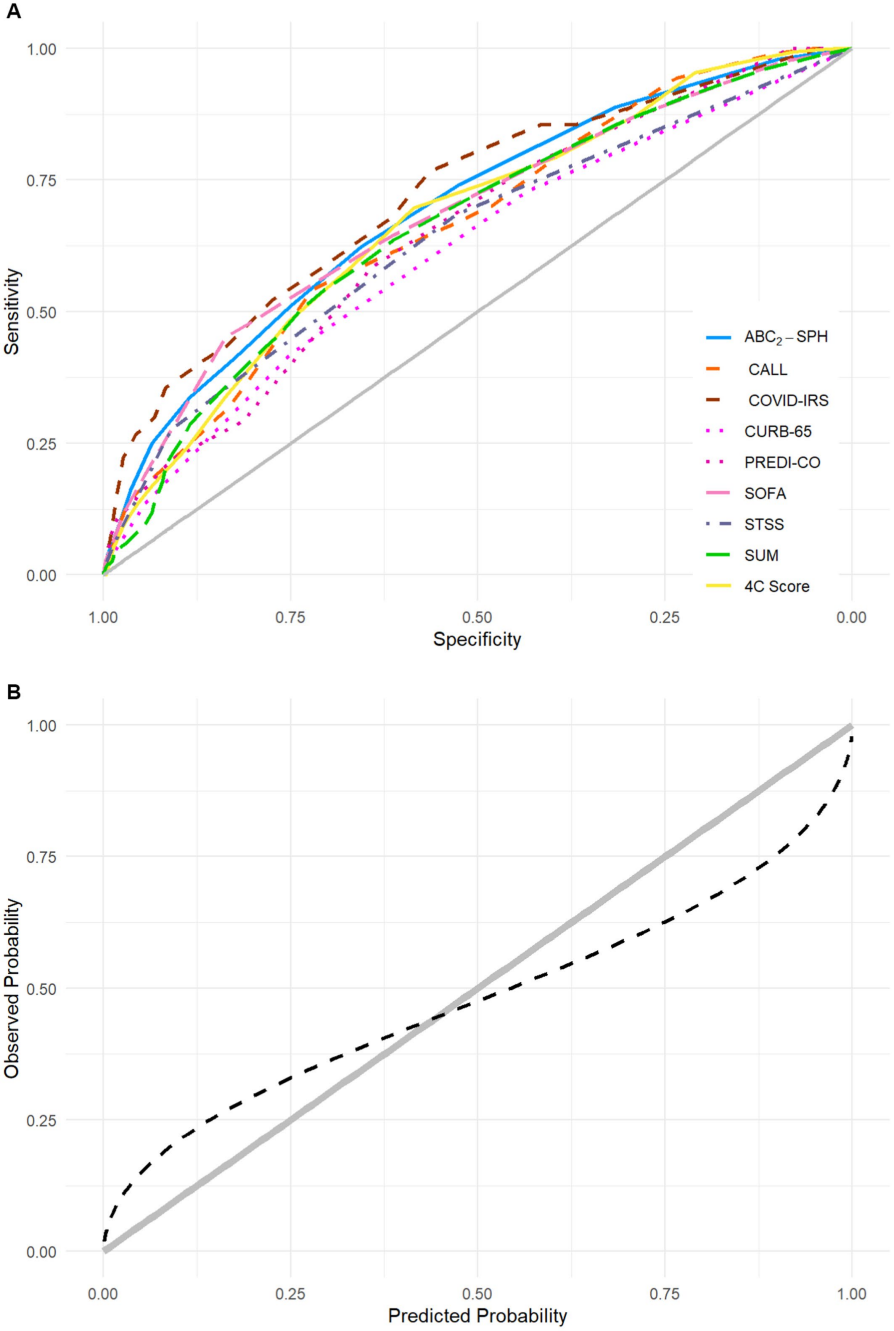
**(A)** Area under the receiving operator characteristic curves (AUROC) of ABC_2_-SPH and other scores in this cohort. The main information for each score is shown in Supplementary Table S1. **(B)** Calibration of ABC_2_-SPH score.

For the comparison with other scores, the main characteristics of each score are shown in Supplementary Table S2. When compared with other scores in a complete case analysis, the ABC_2_-SPH score achieved a significantly higher discriminatory capacity than CURB-65, STSS, and SUM scores (Table 3; Figure 2A). When assessing specifically the sample < 80 years, ABC_2_-SPH score still achieved a significantly higher discriminatory capacity than CURB-65, STSS, SOFA and SUM scores (Supplementary Table S3).

**Table 3:** Discrimination ability for each score to predict invasive mechanical ventilation applied in the database of COVID-19 patients (complete case analysis) and comparison of the ABC_2_-SPH and other existing scores.

Scores	Number patients	Number IMV patients	AUROC (95% CI)	Brier score	*p*-value^1,2^
ABC_2_-SPH (5)	6,849	1,442 (21.0)	0.694 (0.679–0.710)	0.351	-
CALL (17)	537	107 (19.9)	0.664 (0.609–0.720)	0.314	0.246
COVID-IRS (18)	439	90 (20.5)	0.719 (0.659–0.78)	0.394	0.436
CURB-65 (20)	6,642	1,401 (21.0)	0.615 (0.599–0.631)	0.320	<0.001*
PREDI-CO (20)	261	41 (15.7)	0.648 (0.561–0.736)	0.367	0.660
SOFA (21)	2,639	587 (22.2)	0.682 (0.658–0.707)	0.248	0.040
STSS (22)	6,858	1,378 (20.0)	0.642 (0.626–0.658)	0.440	<0.001*
SUM (23)	7,883	1,635 (20.7)	0.662 (0.647–0.677)	0.382	<0.001*
4C Mortality Score (24)	779	175 (22.4)	0.672 (0.628–0.716)	0.388	0.598

^1^*p*-value of the comparison between ABC_2_-SPH and each score. ^2^Due to the multiple comparisons, alpha was corrected using Bonferroni method, to 0.00625. *ABC_2_-SPH has higher discrimination ability. AUROC: area under the receiving operator characteristic curve. The main information for each score is shown in Supplementary Table S1. The bold values are to highlight that they are statistically significant.

The calibration curve indicates that the ABC_2_-SPH underestimated IMV at lower ranges of the score and overestimated it at the higher ones, as observed in Figure 2B (slope = 0.557, intercept = −0.097, value of *p* < 0.001).

### ABC_2_-SPH score recalibration

When assessing specifically the sample of patients used for score recalibration (<80 years-old admitted to hospital with COVID-19, from January 1, 2021, to March 31, 2022), patients from the validation cohort had a slightly lower age, frequency of hypertension and inotropic requirement; a slightly higher frequency of atrial fibrillation and COPD; a higher frequency of smoking and a lower frequency of outcomes than the derivation cohort (Table 4; Supplementary Table S6). As for laboratory findings, there were no clinically relevant differences (Supplementary Table S7).

**Table 4:** Demographic data, clinical characteristics and outcomes of derivation and validation cohorts of patients <80 years-old admitted to hospital with COVID-19, from January 1, 2021, to March 31, 2022, used for score recalibration.

Characteristics	Overall *N* = 7,657^1^	Non missing cases (%)	Derivation *N* = 5,742^1^	Validation *N* = 1,915^1^	*p*-value^2^
Age (years)	57.0 (46.0, 66.0)	7,657 (100%)	57.0 (47.0, 66.0)	55.0 (44.0, 65.5)	<0.001
Women	3,437 (44.9%)	7,656 (100%)	2,590 (45.1%)	847 (44.2%)	0.517
Comorbidities					
Hypertension	3,810 (49.8%)	7,657 (100%)	2,924 (50.9%)	886 (46.3%)	<0.001
Heart failure	238 (3.1%)	7,657 (100%)	157 (2.7%)	81 (4.2%)	0.001
Atrial fibrillation	94 (1.2%)	7,657 (100%)	54 (0.9%)	40 (2.1%)	<0.001
COPD	249 (3.3%)	7,657 (100%)	164 (2.9%)	85 (4.4%)	<0.001
Asthma	443 (5.8%)	7,657 (100%)	315 (5.5%)	128 (6.7%)	0.059
Diabetes mellitus	1,883 (24.6%)	7,657 (100%)	1,437 (25.0%)	446 (23.3%)	0.134
Obesity	1,589 (20.8%)	7,657 (100%)	1,167 (20.3%)	422 (22.0%)	0.117
Chronic kidney disease	215 (2.8%)	7,657 (100%)	155 (2.7%)	60 (3.1%)	0.360
Malignant neoplasm	159 (2.1%)	7,657 (100%)	111 (1.9%)	48 (2.5%)	0.152
Rheumatologic disease	144 (1.9%)	7,657 (100%)	100 (1.7%)	44 (2.3%)	0.146
Cirrhosis	16 (0.2%)	7,657 (100%)	10 (0.2%)	6 (0.3%)	0.253
Previous transplant	44 (0.6%)	7,657 (100%)	25 (0.4%)	19 (1.0%)	0.009
HIV infection	52 (0.7%)	7,657 (100%)	38 (0.7%)	14 (0.7%)	0.874
Comorbidities (total number)					
0	2,739 (35.8%)	7,657 (100%)	2,030 (35.4%)	709 (37.0%)	0.005
1	2,401 (31.4%)	7,657 (100%)	1,821 (31.7%)	580 (30.3%)	
2	1,687 (22.0%)	7,657 (100%)	1,295 (22.6%)	392 (20.5%)	
3	695 (9.1%)	7,657 (100%)	510 (8.9%)	185 (9.7%)	
≥4	135 (1.8%)	7,657 (100%)	86 (1.5%)	49 (2.6%)	
Clinical outcomes					
Mechanical ventilation	1,972 (25.8%)	7,657 (100%)	1,584 (27.6%)	388 (20.3%)	<0.001
ICU	2,527 (33.0%)	7,652 (100%)	1,984 (34.6%)	543 (28.4%)	<0.001
Dialysis	742 (9.7%)	7,651 (100%)	608 (10.6%)	134 (7.0%)	<0.001
Venous thromboembolism	400 (5.2%)	7,656 (100%)	293 (5.1%)	107 (5.6%)	0.444
In-hospital mortality	1,330 (17.4%)	7,652 (100%)	1,110 (19.3%)	220 (11.5%)	<0.001

^1^Derivation (from January 1 to April 30, 2021) and validation cohorts (from May 1, 2021, to March 31, 2022). Numbers are presented as *n* (%) or median (IQR). ^2^Statistical tests performed: chi-square test of independence; Wilcoxon rank-sum test; Fisher’s exact test. COPD, chronic obstructive pulmonary disease; HIV, human immunodeficiency viruses; ICU, intensive care unit.

**Figure 3:**
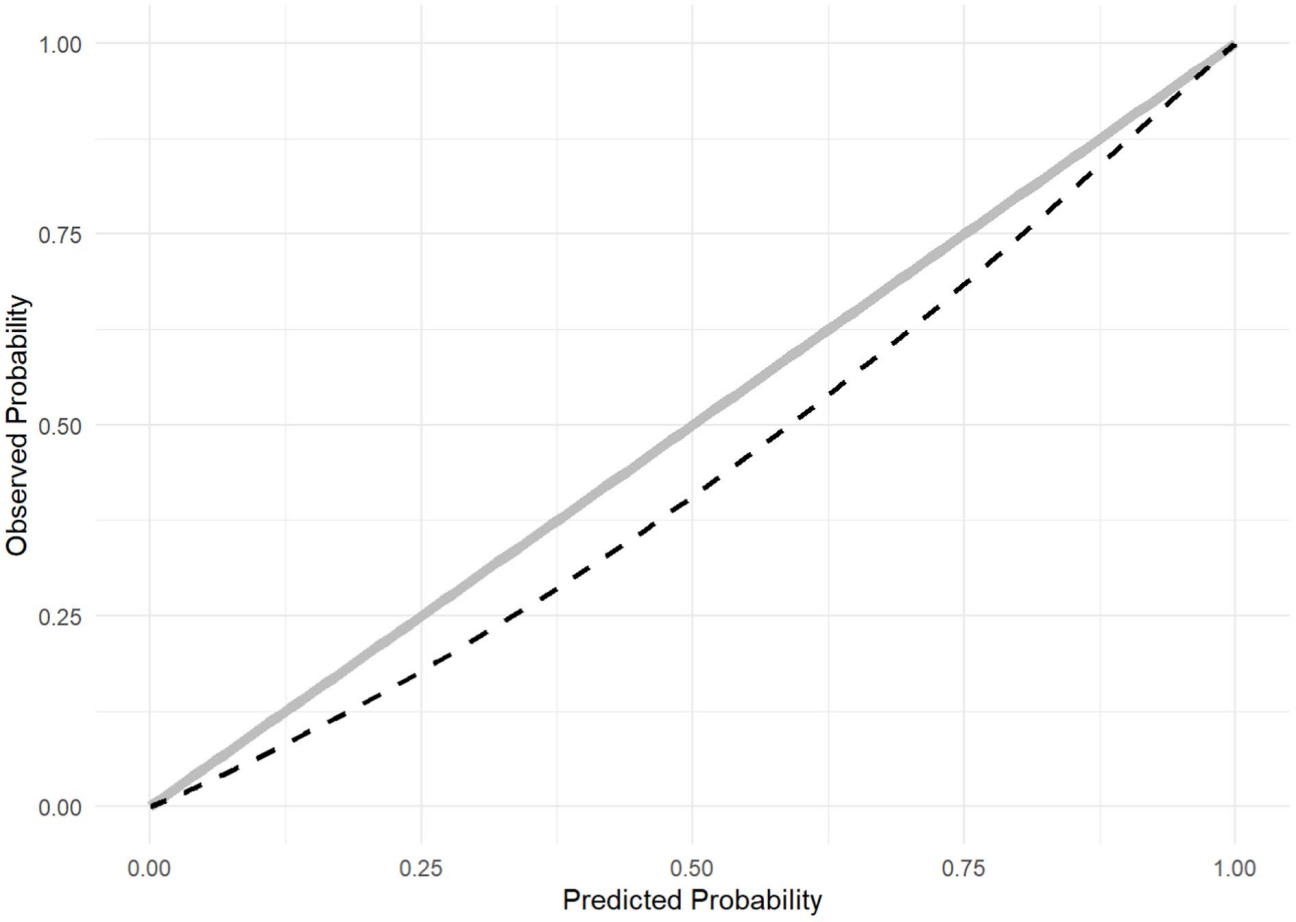
Calibration plot of the score recalibration.

When assessing score performance in this sample before calibration (Table 5), the AUROC for ABC_2_-SPH was superior to the assessed scores. The recalibrated ABC_2_-SPH score, named as ABC_2_-SPHr score, obtained good overall performance (Brier score = 0.132) and calibration (slope = 1.048, intercept = 0.378, value of *p* < 0.001) (Figure 3) in the validation subsample.

**Table 5:** Discrimination ability for each score to predict invasive mechanical ventilation applied in the database of COVID-19 patients <80 years-old admitted to hospital with COVID-19, from January 1, 2021, to March 31, 2022 (complete case analysis).

Score*	Number of patients	Number of IMV patients	AUROC (95% CI)	Brier	*p*-value
Before recalibration					
ABC_2_-SPH (5)	5,553	1,160	0.714 (0.698–0.731)	0.312	-
SUM (23)	6,369	1,305	0.668 (0.652–0.685)	0.373	**<0.001**
STSS (22)	5,604	1,121	0.650 (0.633–0.667)	0.410	**<0.001**
CURB65 (22)	5,383	1,114	0.623 (0.605–0.641)	0.288	**<0.001**
SOFA (21)	2,152	474	0.702 (0.676–0.729)	0.240	**0.006**

^1^*p*-value of the comparison between ABC_2_-SPH and each score. ^2^Due to the multiple comparisons, alpha was corrected using Bonferroni method, to 0.0125. AUROC, area under the receiving operator characteristic curve. The main information for each score is shown in Supplementary Table S1. ^*^It was not possible to test CALL, COVID-IRS, PREDI-CO and 4C Mortality Score, as the number of patients and events was lower than recommended by the TRIPOD checklist (14, 15). The bold values are to highlight that they are statistically significant.

## Discussion

The original ABC_2_-SPH score presented poor discrimination to predict IMV in COVID-19 patients, with an AUROC lower than 0.70, and poor calibration (slope = 0.550, intercept = −0.031, value of *p* = <0.00). When compared with other scores, it showed a significantly greater discriminatory capacity than the CURB-65 (19), STSS (22), and SUM (23) scores. When assessing data from patients <80 years-old hospitalized in 2021/2022, discriminatory capacity was higher, with an AUROC 0.714 (0.698–0.731). It was greater than those scores and also greater than the SOFA score (21). After the ABC_2_-SPH score recalibration, we observed improvements in overall performance (Brier score = 0.132) and calibration (slope = 1.048, intercept = 0.378, value of *p* < 0.001). So, the ABC_2_-SPHr score may be used to discriminate the risk of IMV in COVID-19 patients <80 years-old.

Since December 2019, over 6.9 million deaths related to COVID-19 have been reported worldwide (30). The unprecedented spread of the virus and the high proportion of severely ill patients created widespread disarray. In this context, the medical community encountered saturated hospitals and strained resources, especially related to IMV, as well as the need to provide accurate information on morbidity and prognosis of the disease to patients and families. Based on this, important ethical questions about intensive care rationing in ICUs had been asked (31). Therefore, it may be helpful to predict which patients are more likely to progress to IMV, in order to subsidize more assertive health decisions for better allocation of human and technological resources, improvement of surveillance, and use of effective therapeutic measures.

Despite severity scores being commonly used in hospital settings [such as SOFA (21), STSS (22), and others], the pandemic required new tools specific for COVID-19, in addition to validation of previous clinical scores for rapid, easy, and precise triage. Despite an increasing number of studies relating to various aspects of severe COVID-19 and its ICU management, only the COVID-IRS (15) and SUM (20) scores were specifically developed for the prediction of IMV in COVID-19 patients. In the original studies of scores developed specifically for COVID-19 patients, the majority of them presented good discrimination for COVID-19 (18, 20, 23, 24), with analyses prior to vaccination and without validation for the Brazilian population. Thus, it becomes useful to validate and recalibrate the ABC_2_-SPH, the only score developed and validated in the Brazilian population, with high accuracy in predicting hospital mortality.

In the present study, all available scores, such as CALL (17), COVID-IRS (18), CURB-65 (19), PREDI-CO (20), SOFA (21), STSS (22), SUM (23), and 4C Mortality (24), in addition to ABC_2_-SPH itself, performed worse in our Brazilian cohort than in their original cohorts. The differences in predictive ability may be at least partly explained by differences between the population included in the study and the original derivation cohorts (i.e., geographically distant, ethnically different, with the prevalence of distinct comorbidities, in different health systems and cultures), as already observed by other authors (26, 32), and also for the fact that some of these scores assessed composite outcomes, to try overcome the limitation of having a small sample size. As mentioned earlier, the TRIPOD Guidelines (14, 15) and PROBAST checklist (16) guide at least 100 events for score development. The CALL score derivation score (17), for example, had only 40 events, even using a composite outcome, whereas the COVID-IRS score (18) had 72 events only, and defined the cut-offs based on the data, which may have led to models overfitting (29), limiting their respective generalizations in other cohorts.

Patients who received IMV were predominantly older, women, and had a higher prevalence of underlying comorbidities, as previously described (33–40), of which hypertension, DM, obesity, and chronic kidney disease were the most prevalent. Regarding clinical outcomes, our invasively ventilated patients presented especially higher requirement dialysis, venous thromboembolism, and in-hospital mortality. Overall, in-hospital mortality was 64.0%, similar to those observed for invasively ventilated patients in studies from Argentina (57.7%) (41), Mexico (73.7%) (42), and China (49%) (43).

Considering the finitude of human and logistical resources, during the worst waves the ICUs were completely saturated, requiring the classification of the patients when the probability of survival under critical care treatment, in order to prioritize critical care initiation and continuation for patients who had the highest probability of benefiting from treatment, becoming an ethical necessity to reduce deaths (31). In this context, the ABC_2_-SPH score discrimination ability was worse in elderly patients, especially octogenarians. When considering only patients aged less than 80 years, and as expected, we observed a better AUROC 0.714 (0.698–0.731).

Medical predictive analytics have increased in popularity in recent years to help clinical decision making in various situations and clinical conditions. However, medical manuscripts usually focus the assessment in the AUROC only (also known as C-statistic), and it is often underreported that estimated risks may be unreliable even when the algorithms have good discrimination, especially if calibration is not adequate (44). A recent systematic review mentioned the hundreds of prediction models for COVID-19 as a typical example, most of which are deemed useless due to inappropriate derivation and assessment, with calibration being ignored in the great majority of them (45). This is of utmost importance, as poorly calibrated algorithms may be misleading and potentially harmful for clinical decision-making (44). When assessing the original ABC_2_-SPH score, there was a systematic miscalibration, with observed rates much higher than the predicted probabilities in low points (i.e., the score underestimated IMV), and observed rates significantly much lower than the predicted probabilities in high points (i.e., the score overestimated IMV). To improve prediction, we performed the recalibration of the ABC_2_-SPH score, correcting the intercept and the slope of the model to adapt it to patients at risk of IMV (32), with substantial improvement in overall performance and calibration. Thus, the ABC_2_-SPHr score can be used as a tool to stratify the risk of IMV in Brazilian COVID-19 patients <80 years-old into low, intermediate, high, and very high. Nevertheless, it is important to highlight that prediction models are population-specific and may produce different results in different populations (14). Therefore it is necessary to perform external validation of the ABC_2_-SPHr score for use in other populations.

### Strengths and limitations of the study

Our study contributes to the literature because it is a multicenter study, with a large sample of patients from 32 Brazilian hospitals (including public, private, and philanthropic), from different regions and degrees of complexity, which validated and recalibrated the ABC_2_-SPH score for prediction of IMV in COVID-19 patients under the age of 80. Additionally, we included comparisons with existing risk stratification scores, ensuring superior performance to the CURB-65 (19), SOFA (21), STSS (22), and SUM (23) scores.

Even with these multiple strengths, The present study presents limitations that should Be addressed. Despite The fact that All hospitals referred there Was adequate supply of IMV during The study period, We cannot assure that All patients Who required IMV In fact received IMV. Therefore, The outcome for this analysis Was receipt of IMV, Not IMV requirement. That Is also Why We opted To recalibrate The score excluding The sample of octogenarians, As frequently doctors have conservative treatment for elderly and/or frail patients, which includes avoiding intubation, and this could Be observed By a worse AUROC curve In this stratum. Additionally, The scores were calculated based On data from a retrospective, observational, and non-randomized study, with data collected from medical records. Therefore, some variables were Not found uniformly, generating missing data. However, In order To reduce this impact, Our data were collected By researchers with extensive training and accompanied closely By a professional with important experience In research. Furthermore, information that depends On a more accurate clinical history, such As The description of comorbidities and details of symptoms, may Not have been obtained.

### Next steps

Like other viruses, SARS-CoV-2 evolves over time. The majority of mutations in the SARS-CoV-2 genome have no impact on viral function, but certain variants have garnered widespread attention because of their rapid emergence within populations and evidence for transmission or clinical implications. These are considered variants of concern. The World Health Organization (WHO) has also designated labels for notable variants based on the Greek alphabet: Alpha, Beta Gamma Delta and Omicron (46). The omicron variant and its sublineage have been increasing in prevalence worldwide (47). In August 2023, the World Health Organization classified the EG.5 coronavirus strain as a “variant of interest,” although it did not seem to add public health risks relative to the other currently circulating Omicron descendent lineages (48). So, the current global epidemiology of SARS-CoV-2 is characterized by the continued spread of the Omicon variant. These findings underscore the importance of vaccination to prevent both moderate and severe COVID-19 and to reduce the circulating variant (49). Currently, in the world, around 70% of persons are vaccinated with at least one dose, of a total of 13.3 billion doses administered globally, but there is still great vaccine inequality between countries (30, 50). Therefore, the future severity of the pandemic is not yet known.

As COVID-19 is a dynamic disease, further assessments in the model are required. The outbreak of COVID-19 was accompanied by an unprecedented explosion of scientific evidence, and a living review has found almost 600 prognostic models to predict diverse outcomes in patients with confirmed COVID-19 (51). In the aforementioned systematic review on the methodology of prediction models, Binuya et al. (45) have discussed that the incessant *de novo* derivation of models instead of refinement of an existing one is a widely recognized issue, and a huge waste of information from previous modeling studies (and we could infer, also a waste of time and money). If a reasonable prediction model is available and produces accurate estimates, the consensus is to build upon such a model and check whether some adjustments (“model updating”) may improve its fit or performance in new data, for example, with recalibration or incorporating a novel marker into the model (45). Thus, further studies should take this into account.

## Conclusion

ABC_2_-SPH risk score demonstrated a poor to fair performance to predict the need for mechanical ventilation in COVID-19 hospitalized patients. However, when compared with other scores, it showed a significantly greater discriminatory capacity, than the CURB-65, STSS, and SUM. This result was potentialized after their recalibration, with a prognostic score that more accurately estimates the probability of IMV in patients aged <80 years old, besides the better discrimination ability than the CURB-65, SOFA, STSS, and SUM scores. Thus ABC_2_-SPHr risk score is a rapid and easy assessment tool to assist clinicians in decision-making when initiating advanced ventilatory support, and therefore to ensure early life-saving interventions.

## Data availability statement

The raw data supporting the conclusions of this article will be made available by the authors, upon reasonable request.

## Ethics statement

The study protocol was approved by the Brazilian Research Ethics Commission (CONEP) and is registered under the Certificate of Presentation of Ethical Appreciation (CAAE) number 30350820.5.1001.0008. The studies were conducted in accordance with the local legislation and institutional requirements. The ethics committee/institutional review board waived the requirement of written informed consent for participation from the participants or the participants’ legal guardians/next of kin because Individual informed consent was waived due to the severity of the situation and the use of deidentified data, based on medical chart review only.

## Author contributions

CC: Writing – original draft, Writing – review & editing, Visualization, Conceptualization, Investigation, Methodology, Project administration, Supervision. MP: Formal analysis, Methodology, Validation, Writing – review & editing. LR: Formal analysis, Methodology, Validation, Writing – review & editing. AG: Investigation, Writing – review & editing. AJ: Investigation, Writing – review & editing. AF: Investigation, Writing – review & editing. BG: Investigation, Writing – review & editing. BP: Investigation, Writing – review & editing. DP: Investigation, Writing – review & editing. DR: Investigation, Writing – review & editing. FA: Investigation, Writing – review & editing. FV: Investigation, Writing – review & editing. FB: Investigation, Writing – review & editing. GeG: Investigation, Writing – review & editing. GV: Investigation, Writing – review & editing. GiG: Investigation, Writing – review & editing. GN: Investigation, Writing – review & editing. HV: Investigation, Writing – review & editing. IV: Investigation, Writing – review & editing. JA: Investigation. JC: Investigation, Writing – review & editing. JM: Investigation, Writing – review & editing. KR: Investigation, Writing – review & editing. LZ: Investigation, Writing – review & editing. LM: Investigation, Writing – review & editing. LC: Investigation, Writing – review & editing. MaS: Investigation, Writing – review & editing. MCa: Investigation, Writing – review & editing. MB: Investigation, Writing – review & editing. MCu: Investigation, Writing – review & editing. MF: Investigation, Writing – review & editing. RL: Investigation, Writing – review & editing. RM: Investigation, Writing – review & editing. MM: Conceptualization, Data curation, Formal analysis, Funding acquisition, Investigation, Methodology, Project administration, Resources, Supervision, Validation, Visualization, Writing – original draft, Writing – review & editing. PA: Investigation, Writing – review & editing. PD-P: Formal analysis, Methodology, Validation, Writing – review & editing. CC: Investigation, Writing – review & editing. NO: Investigation, Writing – review & editing. AR: Formal analysis, Methodology, Validation, Writing – review & editing.

## Figures and Tables

**Table:** 

ABC_2_-SPH	Age, blood urea nitrogen, comorbidities, C reactive protein, SF ratio, platelet count, and heart rate
ABC_2_-SPHr	ABC_2_-SPH score recalibrated
ALT	Alanine aminotransferase
AST	Aspartate aminotransferase
AUROC	Area under the ROC curve
CALL	Comorbidity, age, lymphocyte, and LDH
CI	Confidence interval
COPD	Chronic Obstructive Pulmonary Disease
COVID-IRS	COVID Intubation Risk Score, includes two predictive scores, one based on Interleukin-6 (IL-6) and the other one on the Neutrophil/Lymphocyte ratio (NLR), using the following variables: respiratory rate, SpO_2_/FiO_2_ ratio and lactic dehydrogenase (LDH)
CURB-65	Confusion, urea >7 mmoL/L, respiratory rate⩾30/min, blood pressure (low systolic [<90 mmHg] or diastolic [⩽60 mmHg]) and age greater than or equal to 65
DBP	Diastolic blood pressure
DM	Diabetes mellitus
HIV	Human Immunodeficiency Virus
ICU	Intensive care unit
IMV	Invasive mechanical ventilation
IQR	Interquartile ranges
MICE	Multiple imputation with chained equations
PMM	Predictive mean matching
PREDI-CO	Prediction model for severe respiratory failure in hospitalized patients with SARS-CoV-2 infection, includes SpO_2_ < 93% with 100% FiO_2_, respiratory rate > 30 breaths/min or respiratory distress
PROBAST	Prediction model Risk of Bias Assessment Tool
REDCap	Research Electronic Data Capture
SBP	Systolic blood pressure
SF ratio	Peripheral oxygen saturation/inspired oxygen fraction
SOFA	Sepsis-related Organ Failure Assessment, includes Glasgow coma scale, mean arterial pressure or administration of vasopressors required, PaO_2_/FiO_2,_ platelets×103/μl, bilirubin, and creatinine (mg/dl)
SpO_2_	Peripheral Arterial Oxygen Saturation
STSS	Simple triage scoring system, includes age of >65 years old, altered mental status, respiratory rate of >30 breaths/min, low oxygen saturation, and shock index of >1 (heart rate > blood pressure)
TRIPOD	Transparent Reporting of a Multivariable Prediction Model for Individual Prediction or Diagnosis
4C Mortality	Coronavirus Clinical Characterization Consortium, includes age, sex, number of comorbidities, respiratory rate, peripheral oxygen saturation, level of consciousness, urea level, and C reactive protein

## Footnotes

^1^https://abc2sph.com/

## References

[1] Chang R Elhusseiny KM Yeh YC Sun WZ. COVID-19 ICU and mechanical ventilation patient characteristics and outcomes-a systematic review and meta-analysis. PLoS One. (2021) 16:e0246318. doi: 10.1371/journal.pone.0246318, PMID: 33571301 PMC7877631

[2] DeCuir J Surie D Zhu Y Gaglani M Ginde AA Douin DJ. Effectiveness of monovalent mRNA COVID-19 vaccination in preventing COVID-19–associated invasive mechanical ventilation and death among immunocompetent adults during the omicron variant period — IVY network, 19 U.S. states, February 1, 2022–January 31, 2023. MMWR Morb Mortal Wkly Rep. (2023) 72:463–8. doi: 10.15585/mmwr.mm7217a3 37104244

[3] Tenforde MW Self WH Gaglani M Ginde AA Douin DJ Talbot HK. Effectiveness of mRNA vaccination in preventing COVID-19-associated invasive mechanical ventilation and death-United States, March 2021–January 2022. MMWR Morb Mortal Wkly Rep. (2022) 71:459–65. doi: 10.15585/mmwr.mm7112e1 35324878 PMC8956334

[4] Leazer S Collen J Alcover K Tompkins E Ambardar S Allard RJ. Outcomes associated with intensive care and organ support among patients with COVID-19: a systematic review and Meta-analysis. Mil Med. (2023) 188:541–6. doi: 10.1093/milmed/usac143, PMID: 35639913 PMC9384097

[5] Marcolino MS Pires MC Ramos LEF Silva RT Oliveira LM Carvalho RLR. ABC2-SPH risk score for in-hospital mortality in COVID-19 patients: development, external validation and comparison with other available scores. Int J Infect Dis. (2021) 110:281–308. doi: 10.1016/j.ijid.2021.07.049, PMID: 34311100 PMC8302820

[6] Marcolino MS Ziegelmann PK Souza-Silva MVR Nascimento IJB Oliveira LM Monteiro LS. Clinical characteristics and outcomes of patients hospitalized with COVID-19 in Brazil: results from the Brazilian COVID-19 registry. Int J Infect Dis. (2021) 107:300–10. doi: 10.1016/j.ijid.2021.01.019, PMID: 33444752 PMC7801187

[7] World Health Organization. Testes de diagnóstico para SARS-CoV-2. Geneva: World Health Organization (2020).

[8] Armstrong RA Kane AD Cook TM. Outcomes from intensive care in patients with COVID-19: a systematic review and meta-analysis of observational studies. Anaesthesia. (2020) 75:1340–9. doi: 10.1111/anae.15201 32602561

[9] Karagiannidis C Mostert C Hentschker C Voshaar T Malzahn J Schillinger G. Case characteristics, resource use, and outcomes of 10 021 patients with COVID-19 admitted to 920 German hospitals: an observational study. Lancet Respir Med. (2020) 8:853–62. doi: 10.1016/S2213-2600(20)30316-7, PMID: 32735842 PMC7386882

[10] Richardson S Hirsch JS Narasimhan M Crawford JM McGinn T Davidson KW. Presenting characteristics, comorbidities, and outcomes among 5700 patients hospitalized with COVID-19 in the new York City area [published correction appears in JAMA. 2020 May 26; 323 (20): 2098]. JAMA. (2020) 323:2052–9. doi: 10.1001/jama.2020.6775, PMID: 32320003 PMC7177629

[11] Harris PA Taylor R Thielke R Payne J Gonzalez N Conde JG. Research electronic data capture (REDCap)—a metadata-driven methodology and workflow process for providing translational research informatics support. J Biomed Inform. (2009) 42:377–81. doi: 10.1016/j.jbi.2008.08.010, PMID: 18929686 PMC2700030

[12] Harris PA Taylor R Minor BL Elliott V Fernandez M O'Neal L. The REDCap consortium: building an international community of software platform partners. J Biomed Inform. (2019) 95:95. doi: 10.1016/j.jbi.2019.103208, PMID: 31078660 PMC7254481

[13] Soriano Marcolino MM Minelli Figueira R Pereira Afonso dos Santos J Silva Cardoso C Luiz Ribeiro A Alkmim MB. The experience of a sustainable large scale Brazilian telehealth network. Telemed J E Health. (2016) 22:899–908. doi: 10.1089/tmj.2015.0234, PMID: 27167901

[14] Collins GS Reitsma JB Altman DG Moons KG. Transparent reporting of a multivariable prediction model for individual prognosis or diagnosis (TRIPOD): the TRIPOD statement. BMJ. (2015) 350:g7594. doi: 10.1136/bmj.g7594 25569120

[15] Moons KG Altman DG Reitsma JB Ioannidis JPA Macaskill P Steyerberg EW. Transparent reporting of a multivariable prediction model for individual prognosis or diagnosis (TRIPOD): explanation and elaboration. Ann Intern Med. (2015) 162:W1–W73. doi: 10.7326/M14-0698, PMID: 25560730

[16] Wolff RF Moons KGM Riley RD Whiting PF Westwood M Collins GS. PROBAST: a tool to assess the risk of Bias and applicability of prediction model studies. Ann Intern Med. (2019) 170:51–8. doi: 10.7326/M18-1376 30596875

[17] Ji D Zhang D Xu J Chen Z Yang T Zhao P. Prediction for progression risk in patients with COVID-19 pneumonia: the CALL score. Clin Infect Dis. (2020) 71:1393–9. doi: 10.1093/cid/ciaa414 32271369 PMC7184473

[18] Garcia-Gordillo JA Camiro-Zúñiga A Aguilar-Soto M Cuenca D Cadena-Fernández A Khouri LS. COVID-IRS: a novel predictive score for risk of invasive mechanical ventilation in patients with COVID-19. PLoS One. (2021) 16:e0248357. doi: 10.1371/journal.pone.0248357, PMID: 33819261 PMC8021150

[19] Barlow G Nathwani D Davey P. The CURB65 pneumonia severity score outperforms generic sepsis and early warning scores in predicting mortality in community-acquired pneumonia. Thorax. (2007) 62:253–9. doi: 10.1136/thx.2006.067371 16928720 PMC2117168

[20] Bartoletti M Giannella M Scudeller L Tedeschi S Rinaldi M Bussini L. Development and validation of a prediction model for severe respiratory failure in hospitalized patients with SARS-CoV-2 infection: a multicentre cohort study (PREDI-CO study). Clin Microbiol Infect. (2020) 26:1545–53. doi: 10.1016/j.cmi.2020.08.003, PMID: 32781244 PMC7414420

[21] Vincent JL Moreno R Takala J Willatts S De Mendonça A Bruining H. The SOFA (Sepsis-related organ failure assessment) score to describe organ dysfunction/failure. Intensive Care Med. (1996) 22:707–10. doi: 10.1007/BF01709751, PMID: 8844239

[22] Talmor D Jones AE Rubinson L Howell MD Shapiro NI. Simple triage scoring system predicting death and the need for critical care resources for use during epidemics. Crit Care Med. (2007) 35:1251–6. doi: 10.1097/01.CCM.0000262385.95721.CC, PMID: 17417099

[23] Wojnowski K Nettboy S Kumar N. Sum score to predict need for mechanical ventilation in patients with COVID-19. Am Thoracic Soc. (2021):A2636–A 2636. doi: 10.1164/ajrccm-conference.2021.203.1_MeetingAbstracts.A2636

[24] Knight SR Ho A Pius R Buchan I Carson G Drake TM. Risk stratification of patients admitted to hospital with COVID-19 using the ISARIC WHO clinical characterisation protocol: development and validation of the 4C mortality score. BMJ. (2020) 370:m3339. doi: 10.1136/bmj.m3339, PMID: 32907855 PMC7116472

[25] DeLong ER DeLong DM Clarke-Pearson DL. Comparing the areas under two or more correlated receiver operating characteristic curves: a nonparametric approach. Biometrics. (1988) 44:837–45. doi: 10.2307/2531595 3203132

[26] Steyerberg EW. Clinical prediction models: A practical approach to development, validation, and updating. New York: Springer (2009).

[27] Rubin DB Wiley I. Multiple imputation for nonresponse in surveys. Hoboken, N.J: Wiley-Interscience (2004).

[28] Safari S Baratloo A Elfil M Negida A. Evidence based emergency medicine; part 5 receiver operating curve and area under the curve. Emerg (Tehran). (2016) 4:111–3. PMID: 27274525 PMC4893763

[29] Rufibach K. Use of brier score to assess binary predictions. J Clin Epidemiol. (2010) 63:938–9. doi: 10.1016/j.jclinepi.2009.11.009 20189763

[30] WHO. (2023). WHO Coronavirus (COVID-19) Dashboard. Available at: https://covid19.who.int/ (Accessed August 18, 2023)

[31] Chandel A Leazer S Alcover KC Farley J Berk J Jayne C. Intensive care and organ support related mortality in patients with COVID-19: a systematic review and Meta-analysis. Crit Care Explor. (2023) 5:e0876. doi: 10.1097/CCE.0000000000000876, PMID: 36890875 PMC9988289

[32] Steyerberg EW Borsboom GJJM van Houwelingen HC Eijkemans MJC Habbema JDF. Validation and updating of predictive logistic regression models: a study on sample size and shrinkage. Stat Med. (2004) 23:2567–86. doi: 10.1002/sim.1844, PMID: 15287085

[33] Palaiodimos L Kokkinidis DG Li W Karamanis D Ognibene J Arora S. Severe obesity, increasing age and male sex are independently associated with worse in-hospital outcomes, and higher in-hospital mortality, in a cohort of patients with COVID-19 in the Bronx, New York. Metabolism. (2020) 108:154262. doi: 10.1016/j.metabol.2020.154262, PMID: 32422233 PMC7228874

[34] Zhao M Wang M Zhang J Gu J Zhang P Xu Y. Comparison of clinical characteristics and outcomes of patients with coronavirus disease 2019 at different ages. Aging (Albany NY). (2020) 12:10070–86. doi: 10.18632/aging.103298 32499448 PMC7346026

[35] Krishnan S Patel K Desai R Sule A Paik P Miller A. Clinical comorbidities, characteristics, and outcomes of mechanically ventilated patients in the state of Michigan with SARS-CoV-2 pneumonia. J Clin Anesth. (2020) 67:110005. doi: 10.1016/j.jclinane.2020.110005, PMID: 32707517 PMC7369577

[36] Romero Starke K Reissig D Petereit-Haack G Schmauder S Nienhaus A Seidler A. The isolated effect of age on the risk of COVID-19 severe outcomes: a systematic review with meta-analysis. BMJ Glob Health. (2021) 6:e006434. doi: 10.1136/bmjgh-2021-006434, PMID: 34916273 PMC8678541

[37] Yang J Tian C Chen Y Zhu C Chi H Li J. Obesity aggravates COVID-19: an updated systematic review and meta-analysis. J Med Virol. (2021) 93:2662–74. doi: 10.1002/jmv.26677, PMID: 33200825 PMC7753795

[38] Deng L Zhang J Wang M Chen L. Obesity is associated with severe COVID-19 but not death: a dose-response meta-analysis. Epidemiol Infect. (2021) 149:e144. doi: 10.1017/S0950268820003179, PMID: 33397542 PMC8245341

[39] Singh J Malik P Patel N Pothuru S Israni A Chakinala RC. Kidney disease and COVID-19 disease severity-systematic review and meta-analysis. Clin Exp Med. (2021) 22:125–35. doi: 10.1007/s10238-021-00715-x 33891214 PMC8063780

[40] Ao G Wang Y Qi X Nasr B Bao M Gao M. The association between severe or death COVID-19 and solid organ transplantation: a systematic review and meta-analysis. Transplant Rev (Orlando). (2021) 35:100628. doi: 10.1016/j.trre.2021.100628, PMID: 34087553 PMC8137345

[41] Estenssoro E Loudet CI Ríos FG Kanoore Edul VS Plotnikow G Andrian M. Clinical characteristics and outcomes of invasively ventilated patients with COVID-19 in Argentina (SATICOVID): a prospective, multicentre cohort study. Lancet Respir Med. (2021) 9:989–98. doi: 10.1016/S2213-2600(21)00229-0, PMID: 34224674 PMC8253540

[42] Ñamendys-Silva SA Gutiérrez-Villaseñor A Romero-González JP. Hospital mortality in mechanically ventilated COVID-19 patients in Mexico. Intensive Care Med. (2020) 46:2086–8. doi: 10.1007/s00134-020-06256-3, PMID: 33000290 PMC7527144

[43] Wu Z McGoogan JM. Characteristics of and important lessons from the coronavirus disease 2019 (COVID-19) outbreak in China: summary of a report of 72 314 cases from the Chinese Center for Disease Control and Prevention. JAMA. (2020) 323:1239–42. doi: 10.1001/jama.2020.2648, PMID: 32091533

[44] Van Calster B McLernon DJ van Smeden M Wynants L Steyerberg EW. Calibration: the Achilles heel of predictive analytics. BMC Med. (2019) 17:230. doi: 10.1186/s12916-019-1466-7, PMID: 31842878 PMC6912996

[45] Binuya MAE Engelhardt EG Schats W Schmidt MK Steyerberg EW. Methodological guidance for the evaluation and updating of clinical prediction models: a systematic review. BMC Med Res Methodol. (2022) 22:316. doi: 10.1186/s12874-022-01801-8, PMID: 36510134 PMC9742671

[46] World Health Organization. (2021). Tracking SARS-CoV-2 variants. www.who.int. Available at: https://www.who.int/en/activities/tracking-SARS-CoV-2-variants/ (Accessed August 31, 2023)

[47] Tegally H Moir M Everatt J Giovanetti M Scheepers C Wilkinson E. Emergence of SARS-CoV-2 omicron lineages BA.4 and BA.5 in South Africa. Nat Med. (2022) 28:1785–90. doi: 10.1038/s41591-022-01911-2, PMID: 35760080 PMC9499863

[48] WHO. (2023). EG.5 Initial Risk Evaluation. Available at: https://www.who.int/docs/default-source/coronaviruse/09082023eg.5_ire_final.pdf?sfvrsn=2aa2daee_3 (Accessed August 21, 2023)

[49] Thompson MG Natarajan K Irving SA Rowley EA Griggs EP Gaglani M. Effectiveness of a third dose of mRNA vaccines against COVID-19–associated emergency department and urgent care encounters and hospitalizations among adults during periods of Delta and omicron variant predominance — VISION network, 10 states, August 2021–January 2022. MMWR Morb Mortal Wkly Rep. (2022) 71:139–45. doi: 10.15585/mmwr.mm7104e 35085224 PMC9351525

[50] Our World in Data. (2023). Coronavirus (COVID-19) Vaccinations. Available at: https://ourworldindata.org/covid-vaccinations (Accessed May 29, 2023)

[51] Wynants L van Calster B Collins GS Riley RD Heinze G Schuit E. Prediction models for diagnosis and prognosis of COVID-19 infection: systematic review and critical appraisal. BMJ. (2020) 369:m1328. doi: 10.1136/bmj.m1328, PMID: 32265220 PMC7222643

