# Supplementary material for: Assessment of the ABC2-SPH risk score to predict invasive mechanical ventilation in COVID-19 patients and comparison to other scores
Source: Front Med (Lausanne). 2023 Nov 16;10:1259055. doi: 10.3389/fmed.2023.1259055 (PMC10690599; doi:10.3389/fmed.2023.1259055)
Supplement: Supplementary file 1 [file Table_1.docx]

**SUPPLEMENTARY MATERIAL**

**Table S1.** Discrimination ability of the ABC2-SPH score stratified by age groups, sex and presence or absence of key comorbidities

|  | **AUROC (95% CI)** | **Brier score** |
| --- | --- | --- |
| *Sex* |  |  |
| Men | 0.689 (0.673-0.705) | 0.192 |
| Women | 0.671 (0.652-0.689) | 0.179 |
| *Age (years)* |  |  |
| < 60 | 0.709 (0.691-0.726) | 0.165 |
| 60-69 | 0.704 (0.680-0.728) | 0.211 |
| 70-79 | 0.656 (0.626-0.686) | 0.201 |
| ≥ 80 | 0.646 (0.602-0.689) | 0.215 |
| *Comorbidities* |  |  |
| Hypertension |  |  |
| Present | 0.651 (0.634-0.667) | 0.206 |
| Absent | 0.698 (0.680-0.716) | 0.165 |
| Heart failure |  |  |
| Present | 0.657 (0.597-0.717) | 0.206 |
| Absent | 0.681 (0.669-0.693) | 0.185 |
| COPD |  |  |
| Present | 0.568 (0.505-0.631) | 0.223 |
| Absent | 0.685 (0.673-0.697) | 0.185 |
| Diabetes mellitus |  |  |
| Present | 0.638 (0.615-0.662) | 0.223 |
| Absent | 0.685 (0.671-0.699) | 0.174 |
| Obesity |  |  |
| Present | 0.700 (0.675-0.725) | 0.223 |
| Absent | 0.673 (0.659-0.687) | 0.177 |

COPD: chronic pulmonary obstructive disease

| **Table S2.** Main characteristics of the scores. | | | | |  |
| --- | --- | --- | --- | --- | --- |
| **Score (year)** | **Author** | **Patient time span** | **Country of derivation** | **Sample size (n)** | **Population** |
| ABC_2_-SPH^1^  (2021) | Marcolino et al., | March-September, 2020 | Brazil | 5,506 | Consecutive patients (≥ 18 years) with confirmed COVID-19 admitted to the participating hospitals |
| CALL^2^  (2020) | Ji et al., | 20 January and 22 February 2020 | China | 208 | Consecutive patients with COVID-19 admitted to Fuyang Second People's Hospital or the Fifth Medical Center of Chinese PLA General Hospital |
| COVID-IRS^3^  (2021) | Garcia-Gordillo et al., | March 12, to August 10, 2020 | Mexico | 401 | All COVID-19 patients aged 18 years or older admitted to the American British Cowdray Medical Center |
| CURB 65^4^  (2003) | Barlow et al., | November to April 2001/02 and 2002/03 | UK^7^, New Zeland, the Netherlands | 419 | Patients who receiving antibiotics for a suspected lower respiratory tract infection and had either a new infiltrate on the chest radiograph or had been clinically diagnosed as having community‐acquired pneumonia by a specialist registrar or consultant doctor |
| PREDI-CO^5^  (2020) | Bartoletti et al., | 22 February to 3 April 2020 | Italy | 1,113 | Consecutive adults (≥18 years) diagnosed with COVID-19 |
| SOFA^6^  (1996) | Vincent et al., | (i) Information not available; (ii) May, 1995 | Information not available | (i) 1,643; (ii) Information not available | (i) Information not available; (ii) all patients admitted to the ICU throughout the month of May 1995, except for those staying for  less than 48 h for elective surgery (routine postoperative  surveillance). |
| STSS^7^  (2007) | Talmor et al., | February 1, 2000, and February 1, 2001; December 10, 2003,  and September 30, 2004; between July 2004 and June 2005 | USA | 5,133 | Consecutive adult patients (>18 yrs old) presenting with and without suspected infection. |
| SUM^8^  (2021) | Wojnowski et al., | Information not available | USA | 150 | Patients to present to a large, tertiary referral center in the Southeastern US with COVID-19 pneumonia |
| 4C Mortality Score^9^  (2020) | Knight et al., | To predict mortality in patients admitted to hospital with COVID-19 | England, Scotland, Wales | 35,463 | Consecutive patients aged 18 years and older with a completed index admission to one of 260 hospitals |

| **Table S2b.** Main characteristics of the scores. | | | | | |
| --- | --- | --- | --- | --- | --- |
| **Score (year)** | **Specially developed** | **Methods for development** | **Variables** | **External validation** | **Limitations** |
| ABC_2_-SPH^1^  (2021) | To predict in-hospital mortality in COVID-19 patients | Logistic regression analysis | Age, blood urea nitrogen, comorbidities, C reactive protein, SF ratio, platelet count, and heart rate | Yes | Regional differences of treatment and lab tests, unavailability of some laboratory parameters |
| CALL^2^  (2020) | Prediction for Progression Risk in Patients With COVID-19 Pneumonia | Multivariate Cox regression | Comorbidities, age, lymphocyte, and LDH | NA | Small sample size. A prospective score study is needed to confirm reliability |
| COVID-IRS^3^  (2021) | To predict the risk of invasive mechanical ventilation in patients with COVID-19 | Multivariate logistic regression | Interleukin-6 or NLR and respiratory rate, SpO_2_/FiO_2_ ratio and LDH | Yes | Some patients received different medications prior to hospitalization |
| CURB 65^4^  (2003) | To stratify adults with community acquired pneumonia into different management groups | Not applicable | Confusion, urea, respiratory rate, blood pressure, and age | Yes | Developed for community pneumonia |
| PREDI-CO^5^  (2020) | To predict severe  respiratory failure in hospitalized patients with COVID-19 | Multivariable logistic regression models | Age, obesity, fever at hospitalization, respiratory rate, lymphocyte count, creatinine, C-reactive protein and LDH | Yes | May have been affected by local policies, developed and validated only in Italian hospitals |
| SOFA^6^  (1996) | To describe a sequence of complications in the critically ill, and not to predict outcome | Not applicable | Glasgow coma scale, mean arterial pressure or administration of vasopressors required, PaO_2_/FiO_2_, platelets, bilirubin, and creatinine. | Yes | Information not available |
| STSS^7^  (2007) | To predict death and the need for intensive care unit during an epidemic | Not applicable | Age, mental status, respiratory rate, oxygen saturation and shock index | Yes | Developed for avian influenza, may perform differently for COVID. Sample average age: 54-64 years, and it is known that old age is a predictor of mortality |
| SUM^8^  (2021) | To predict need for mechanical ventilation in patients with COVID-19 | Multivariable logistic regression | Number of comorbidities, NLR, and SpO_2_/FiO_2_ ratio | No | Information not available |
| 4C Mortality Score^9^  (2020) | To predict mortality in patients admitted to hospital with COVID-19 | Logistic regression, machine learning | Age, sex, number of comorbidities, respiratory rate, peripheral oxygen saturation, level of consciousness, urea level, and C reactive protein. | Yes | Important comorbidities (hypertension, previous myocardial  infarction and stroke) were not included in data collection. The derivation cohort comprised patients admitted to hospital who were seriously ill (mortality 32.2%) and were of advanced age (median age 73 years). |
| LDH: lactic dehydrogenase; NA: information not available; NLR: neutrophil/Lymphocyte ratio; ROC: receiving operator characteristic | | | | | |

**References**

^1^Marcolino MS, Pires MC, Ramos LEF, et al. ABC2-SPH risk score for in-hospital mortality in COVID-19 patients: development, external validation and comparison with other available scores. Int J Infect Dis. 2021;110:281-308. doi:10.1016/j.ijid.2021.07.049.

^2^Ji D, Zhang D, Xu J, et al. Prediction for Progression Risk in Patients With COVID-19 Pneumonia: The CALL Score. Clinical Infectious Diseases. 2020;71(6):1393–1399. https://doi.org/10.1093/cid/ciaa414

^3^Garcia-Gordillo JA, Camiro-Zúñiga A, Aguilar-Soto M, et al. COVID-IRS: A novel predictive score for risk of invasive mechanical ventilation in patients with COVID-19. PLoS One. 2021;16(4):e0248357. Published 2021 Apr 5. doi:10.1371/journal.pone.0248357.

^4^Barlow G, Nathwani D, Davey P. The CURB65 pneumonia severity score outperforms generic sepsis and early warning scores in predicting mortality in community-acquired pneumonia. Thorax [Internet]. 2007. 62(3):253–9. doi: 10.1136/thx.2006.067371.

^5^Bartoletti M, Giannella M, Scudeller L, et al. Development and validation of a prediction model for severe respiratory failure in hospitalized patients with SARS-CoV-2 infection: a multicentre cohort study (PREDI-CO study). Clinical Microbiology and Infection. 2020. 26(11):1545e-1553. doi:10.1016/j.cmi.2020.08.003.

^6^Vincent JL, Moreno R, Takala J, et al. The SOFA (Sepsis-related Organ Failure Assessment) score to describe organ dysfunction/failure. Intensive Care Med. 1996. 22:707–710. doi: 10.1007/BF01709751.

^7^Talmor D, Jones AE, Rubinson L, Howell MD, Shapiro NI. Simple triage scoring system predicting death and the need for critical care resources for use during epidemics. Crit Care Med. 2007;35(5):1251-1256. doi:10.1097/01.CCM.0000262385.95721.CC.

^8^Wojnowski K, Nettboy S, Kumar N, et al. Sum Score to Predict Need for Mechanical Ventilation in Patients with COVID-19. American Thoracic Society. 2021;A2636 - A2636. 10.1164/ajrccm-conference.2021.203.1_MeetingAbstracts.A2636

^9^Knight SR, Ho A, Pius R, et al. Risk stratification of patients admitted to hospital with covid-19 using the ISARIC WHO Clinical Characterisation Protocol: development and validation of the 4C Mortality Score. BMJ [Internet]. 2020. 9;370:m3339. doi: 10.1136/bmj.m3339.

| **Table S3.** Comparison of the ABC_2_-SPH score and other existing scores, in COVID-19 patients with <80 years old. | | |
| --- | --- | --- |
| **Compared score** | **p-value** | **AUROC*^1^*** |
| CALL [18] | 0.190 | 0.678 |
| COVID-IRS [19] | 0.468 | 0.746 |
| CURB-65 [20] | **<0.001** | 0.624 |
| PREDI-CO [21] | 0.496 | 0.668 |
| SOFA [22] | **0.005** | 0.689 |
| STSS [23] | **<0.001** | 0.646 |
| SUM [24] | **<0.001** | 0.672 |
| 4C Score [25] | 0.624 | 0.702 |
| ^1^ AUROC of the score used for comparison with ABC_2_-SPH. The main information for each score is shown in Table S1 | | |

| **Table S4.** Self-reported symptoms, toxic habits, clinical assessment and medications of cohort of Brazilian patients admitted to hospital with COVID-19. | | | | | |
| --- | --- | --- | --- | --- | --- |
| **Characteristics** | **Overall**  **N= 9,350*^1^*** | **Non-missing cases** | **IMV**  **N= 2,361*^1^*** | **No IMV**  **N= 6,989*^1^*** | **p-value*^2^*** |
| ***Self-reported symptoms*** | | | | | |
| Duration of symptoms (days) | 8.0 (5.0, 11.0) | 9,283 (99%) | 7.0 (5.0, 10.0) | 8.0 (6.0, 11.0) | <0.001 |
| Dyspnoea | 6,106 (65.3%) | 9,350 (100%) | 1,631 (69.1%) | 4,475 (64.0%) | <0.001 |
| Cough | 5,664 (60.6%) | 9,350 (100%) | 1,371 (58.1%) | 4,293 (61.4%) | 0.004 |
| Fever | 4,913 (52.5%) | 9,350 (100%) | 1,174 (49.7%) | 3,739 (53.5%) | 0.002 |
| Adynamia | 2,121 (22.7%) | 9,350 (100%) | 539 (22.8%) | 1,582 (22.6%) | 0.868 |
| Diarrhoea | 1,569 (16.8%) | 9,350 (100%) | 374 (15.8%) | 1,195 (17.1%) | 0.167 |
| Rhinorrhoea | 1,150 (12.3%) | 9,350 (100%) | 262 (11.1%) | 888 (12.7%) | 0.043 |
| Myalgia | 3,187 (34.1%) | 9,350 (100%) | 711 (30.1%) | 2,476 (35.4%) | <0.001 |
| Headache | 2,167 (23.2%) | 9,350 (100%) | 429 (18.2%) | 1,738 (24.9%) | <0.001 |
| Ageusia | 893 (9.6%) | 9,350 (100%) | 201 (8.5%) | 692 (9.9%) | 0.052 |
| Anosmia | 1,002 (10.7%) | 9,350 (100%) | 223 (9.4%) | 779 (11.1%) | 0.023 |
| Arthralgia | 113 (1.2%) | 9,350 (100%) | 11 (0.5%) | 102 (1.5%) | <0.001 |
| Nausea | 1,284 (13.7%) | 9,350 (100%) | 274 (11.6%) | 1,010 (14.5%) | <0.001 |
| ***Toxic habits*** | | | | | |
| Smoking | 1,733 (18.5%) | 9,350 (100%) | 486 (20.6%) | 1,247 (17.8%) | 0.003 |
| ***Clinical assessment*** | | | | | |
| Temperature (ºC) | 36.4 (36.0, 36.9) | 4,602 (49%) | 36.5 (36.0, 37.1) | 36.4 (36.0, 36.8) | <0.001 |
| Heart rate (bpm^*^) | 85.0 (76.0, 96.0) | 8,252 (88%) | 88.0 (78.0, 99.0) | 85.0 (76.0, 95.0) | <0.001 |
| Respiratory rate (bpm^**^) | 20.0 (18.0, 24.0) | 7,315 (78%) | 24.0 (20.0, 28.0) | 20.0 (18.0, 24.0) | <0.001 |
| State of consciousness: alert | 6,602 (70.6%) | 9,350 (100%) | 1,326 (56.2%) | 5,276 (75.5%) | <0.001 |
| Systolic blood pressure (SBP) | |  |  |  | <0.001 |
| SBP ≥ 90 mmHg | 8,037 (98.3%) | 8,171 (87%) | 1,631 (96.9%) | 6,406 (98.7%) |  |
| SBP < 90 mmHg | 86 (1.1%) | 8,171 (87%) | 26 (1.5%) | 60 (0.9%) |  |
| Inotropic requirement | 49 (0.6%) | 8,172 (87%) | 26 (1.5%) | 23 (0.4%) |  |
| Diastolic blood pressure (DBP) | |  |  |  | <0.001 |
| DBP > 60 mmHg | 7,217 (88.7%) | 8,138 (87%) | 1,433 (85.5%) | 5,784 (89.5%) |  |
| DBP ≤ 60 mmHg | 873 (10.7%) | 8,138 (87%) | 218 (13.0%) | 655 (10.1%) |  |
| Inotropic requirement | 49 (0.6%) | 8,139 (87%) | 26 (1.6%) | 23 (0.4%) |  |
| SF ratio | 350.0 (290.6, 438.1) | 8,423 (90%) | 317.9 (176.0, 423.8) | 381.0 (296.9, 438.1) | <0.001 |
| Glasgow coma score < 15 | 424 (4.5%) | 9,350 (100%) | 132 (5.6%) | 292 (4.2%) | 0.005 |
| ***Medications*** | | | | | |
| Anticoagulants | 386 (4.1%) | 9,350 (100%) | 117 (5.0%) | 269 (3.8%) | 0.023 |
| Corticoids | 161 (1.7%) | 9,350 (100%) | 44 (1.9%) | 117 (1.7%) | 0.603 |
| Oral corticoids | 182 (1.9%) | 9,350 (100%) | 65 (2.8%) | 117 (1.7%) | 0.001 |
| Immunosuppressor | 157 (1.7%) | 9,350 (100%) | 51 (2.2%) | 106 (1.5%) | 0.044 |
| ^1^Statistics presented: n (%); median (IQR). ^2^Statistical tests performed: chi-square test of independence; Wilcoxon rank-sum test; Fisher's exact test. Bpm^*^: beats per minute; Bpm^**^: breaths per minute; SF ratio: peripheral oxygen saturation/inspired oxygen fraction. | | | | | |

|  | | | | | |
| --- | --- | --- | --- | --- | --- |
| **Table S5.** Laboratory findings of cohort of Brazilian patients admitted to hospital with COVID-19. | | | | | |
| **Hemogram** | **Overall**  **N= 9,350*^1^*** | **Non-missing cases** | **IMV**  **N= 2,361*^1^*** | **No IMV**  **N= 6,989*^1^*** | **p-value*^2^*** |
| Haemoglobin (g/L) | 13.4 (12.3, 14.5) | 8,124 (87%) | 13.4 (12.2, 14.6) | 13.4 (12.3, 14.5) | 0.567 |
| Leukocytes count (cels/mm^3^) | 7,415.0 (5,560.0, 9,900.0) | 8,133 (87%) | 7,555.0 (5,542.5, 10,400.0) | 7,400.0 (5,560.0, 9,800.0) | 0.086 |
| Neutrophiles (cels/mm^3^) | 5,616.8 (3,970.0, 7,917.0) | 7,949 (85%) | 6,041.5 (4,202.2, 8,522.0) | 5,544.0 (3,899.0, 7,764.5) | <0.001 |
| Lymphocytes (cels/mm^3^) | 986.5 (698.4, 1,372.0) | 7,943 (85%) | 834.0 (598.0, 1,183.0) | 1,025.0 (732.0, 1,420.0) | <0.001 |
| Platelet count (cels/mm³) | 206,000.0 (160,000.0, 266,000.0) | 8,097 (87%) | 183,000.0 (144,000.0, 230,250.0) | 213,000.0 (165,000.0, 274,000.0) | <0.001 |
| ***Other laboratory results*** | | | | | |
| C-reactive protein (mg/L) | 82.5 (41.4, 139.5) | 7,425 (79%) | 103.1 (60.0, 178.6) | 76.3 (38.0, 127.9) | <0.001 |
| D-dimer | 337.0 (0.9, 980.0) | 4,842 (52%) | 510.0 (1.1, 1,160.0) | 300.0 (0.9, 930.5) | <0.001 |
| AST (U/L) | 44.0 (31.0, 65.7) | 4,686 (50%) | 50.0 (36.9, 74.9) | 42.0 (30.0, 63.0) | <0.001 |
| ALT (U/L) | 39.0 (24.0, 65.0) | 4,652 (50%) | 38.0 (25.0, 62.0) | 39.0 (24.0, 67.0) | 0.589 |
| pH | 7.4 (7.4, 7.5) | 6,866 (73%) | 7.4 (7.4, 7.5) | 7.4 (7.4, 7.5) | <0.001 |
| PO_2_ | 71.0 (61.0, 88.0) | 6,840 (73%) | 67.9 (57.2, 82.4) | 72.0 (62.1, 89.0) | <0.001 |
| PCO_2_ | 35.0 (31.4, 38.4) | 6,838 (73%) | 34.2 (31.0, 38.1) | 35.0 (31.6, 38.5) | 0.002 |
| Bicarbonate | 23.4 (21.5, 25.3) | 6,839 (73%) | 23.0 (20.8, 25.0) | 23.7 (21.8, 25.5) | <0.001 |
| Sodium (mmol/L) | 137.0 (135.0, 140.0) | 7,365 (79%) | 137.0 (134.0, 140.0) | 137.1 (135.0, 140.0) | <0.001 |
| Creatinine (mg/dL) | 0.9 (0.7, 1.1) | 7,872 (84%) | 0.9 (0.8, 1.3) | 0.9 (0.7, 1.1) | <0.001 |
| INR | 1.1 (1.0, 1.1) | 4,483 (48%) | 1.1 (1.0, 1.2) | 1.1 (1.0, 1.1) | <0.001 |
| Lactate | 1.5 (1.1, 2.0) | 5,513 (59%) | 1.5 (1.2, 2.2) | 1.4 (1.1, 1.9) | <0.001 |
| Urea (mg/dL) | 55.0 (39.0, 92.4) | 7,792 (83%) | 53.0 (37.3, 80.8) | 0.044 | 0.044 |
| ^1^Statistics presented: n (%); median (IQR). ^2^Statistical tests performed: chi-square test of independence; Wilcoxon rank-sum test; Fisher's exact test. INR: international normalized ratio; pH: potential of hydrogen; PCO_2_: Partial pressure of carbon dioxide; PO_2_: partial pressure of oxygen; AST: aspartate aminotransferase; ALT: alanine aminotransferase. | | | | | |

| **Table S6**. Clinical assessment, toxic habits, and medications of derivation and validation cohorts of patients <80 years-old admitted to hospital with COVID-19, from January 1, 2021, to March 31, 2022, used for score recalibration. | | | | | |
| --- | --- | --- | --- | --- | --- |
| **Characteristics** | **Overall**  **N= 7,657*^1^*** | **Non-missing cases** | **Derivation**  **N= 5,742*^1^*** | **Validation**  **N= 1,915*^1^*** | **p-value*^2^*** |
| ***Clinical assessment*** | | | | | |
| Heart rate (bpm^*^) | 85.0 (76.0, 96.0) | 7,451 (97%) | 85.0 (76.0, 95.0) | 87.0 (78.0, 98.0) | <0.001 |
| Respiratory rate (bpm^**^) | 21.0 (19.0, 25.0) | 6,692 (87%) | 21.0 (19.0, 25.0) | 20.0 (18.0, 24.0) | 0.009 |
| Systolic blood pressure (SBP) | |  |  |  | 0.001 |
| SBP ≥ 90 mmHg | 7,058 (96.2%) | 7,320 (96%) | 5,269 (95.9%) | 1,789 (96.9%) |  |
| SBP < 90 mmHg | 70 (1.0%) | 7,320 (96%) | 46 (0.8%) | 24 (1.3%) |  |
| Inotropic requirement | 211 (2.9%) | 7,339 (96%) | 178 (3.2%) | 33 (1.8%) |  |
| SF ratio | 257.1 (160.8, 330.5) | 6,310 (82%) | 250.3 (154.6, 324.3) | 276.2 (180.7, 342.9) | <0.001 |
| Glasgow coma score < 15 | 286 (3.7%) | 7,163 (94%) | 207 (3.6%) | 79 (4.1%) | 0.332 |
| ***Toxic habits*** | | | | | |
| Smoking | 1,366 (17.8%) | 7,657 (100%) | 949 (16.5%) | 417 (21.8%) | <0.001 |
| ***Medications*** | | | | | |
| Anticoagulants | 227 (3.0%) | 7,657 (100%) | 167 (2.9%) | 60 (3.1%) | 0.671 |
| Corticoids | 118 (1.5%) | 7,657 (100%) | 85 (1.5%) | 33 (1.7%) | 0.522 |
| Oral corticoids | 116 (1.5%) | 7,657 (100%) | 76 (1.3%) | 40 (2.1%) | 0.023 |
| Immunosuppressor | 118 (1.5%) | 7,657 (100%) | 77 (1.3%) | 41 (2.1%) | 0.019 |
| ^1^Statistics presented: n (%); median (IQR). ^2^Statistical tests performed: chi-square test of independence; Wilcoxon rank-sum test; Fisher's exact test. bpm^*^: beats per minute; bpm^**^: breaths per minute; SF ratio: peripheral oxygen saturation/inspired oxygen fraction. | | | | | |

| **Table S7**. Laboratory findings of derivation and validation cohorts of <80 years-old admitted to hospital with COVID-19, from January 1, 2021, to March 31, 2022, used for score recalibration. | | | | | |
| --- | --- | --- | --- | --- | --- |
| **Characteristics** | **Overall**  **N= 7,657*^1^*** | **Non-missing cases** | **Derivation**  **N= 5,742*^1^*** | **Validation**  **N= 1,915*^1^*** | **p-value*^2^*** |
| ***Laboratory findings*** | | | | | |
| Hemoglobin (g/L) | 13.4 (12.3, 14.5) | 7,315 (96%) | 13.4 (12.3, 14.5) | 13.4 (12.3, 14.5) | 0.949 |
| Leukocytes count (cels/mm^3^) | 7,740.0 (5,770.0, 10,390.0) | 7,325 (96%) | 7,739.5 (5,800.0, 10,390.0) | 7,740.0 (5,670.0, 10,365.0) | 0.489 |
| Neutrophiles (cels/mm^3^) | 5,911.5 (4,221.0, 8,393.0) | 7,180 (94%) | 5,936.0 (4,247.0, 8,420.0) | 5,847.0 (4,083.0, 8,329.0) | 0.174 |
| Lymphocytes (cels/mm^3^) | 976.0 (689.0, 1,365.0) | 7,192 (94%) | 973.0 (688.0, 1,350.0) | 993.0 (689.5, 1,405.0) | 0.087 |
| Platelet count (cels/mm³) | 211,000.0 (164,000.0, 273,000.0) | 7,297 (95%) | 215,000.0 (167,000.0, 278,000.0) | 198,000.0 (154,000.0, 254,000.0) | <0.001 |
| C-reactive protein (mg/L) | 87.6 (46.0, 143.4) | 6,597 (86%) | 87.1 (46.1, 144.1) | 89.2 (45.7, 141.1) | 0.656 |
| D-dimer | 170.4 (0.9, 906.0) | 4,386 (57%) | 65.6 (0.9, 878.5) | 346.0 (0.9, 989.5) | 0.109 |
| AST (U/L) | 46.0 (32.4, 70.0) | 4,367 (57%) | 47.0 (33.0, 71.0) | 44.0 (31.0, 66.0) | <0.001 |
| ALT (U/L) | 41.0 (26.0, 68.0) | 4,345 (57%) | 42.0 (27.0, 70.0) | 38.0 (24.0, 64.0) | <0.001 |
| pH | 7.4 (7.4, 7.5) | 6,351 (83%) | 7.4 (7.4, 7.5) | 7.4 (7.4, 7.5) | 0.018 |
| PO_2_ | 71.7 (61.0, 89.0) | 6,332 (83%) | 71.5 (61.0, 89.1) | 72.0 (62.2, 89.0) | 0.202 |
| PCO_2_ | 35.2 (31.9, 39.0) | 6,333 (83%) | 35.1 (31.7, 39.0) | 35.6 (32.0, 39.4) | 0.019 |
| Bicarbonate | 23.6 (21.6, 25.5) | 6,323 (83%) | 23.4 (21.4, 25.5) | 23.9 (21.9, 25.8) | <0.001 |
| Sodium (mmol/L) | 138.0 (135.0, 140.0) | 6,656 (87%) | 138.0 (135.0, 140.0) | 138.0 (135.0, 140.0) | 0.886 |
| Creatinine (mg/dL) | 0.9 (0.7, 1.1) | 7,114 (93%) | 0.9 (0.7, 1.1) | 0.9 (0.7, 1.1) | 0.064 |
| ^1^Statistics presented: n (%); median (IQR). ^2^Statistical tests performed: chi-square test of independence. pH: potential of hydrogen; PCO_2_: partial pressure of carbon dioxide; PO_2_: partial pressure of oxygen; AST: aspartate aminotransferase; ALT: alanine aminotransferase. | | | | | |
